# Development and validation of a radiomics model of magnetic resonance for predicting liver metastasis in resectable pancreatic ductal adenocarcinoma patients

**DOI:** 10.1186/s13014-023-02273-w

**Published:** 2023-05-10

**Authors:** Yuzhou Huang, Shurui Zhou, Yanji Luo, Jinmao Zou, Yaqing Li, Shaojie Chen, Ming Gao, Kaihong Huang, Guoda Lian

**Affiliations:** 1 Department of Gastroenterology, Sun Yat-Sen Memorial Hospital, Sun Yat-Sen University, Guangzhou, 510120 China; 2 Guangdong Provincial Key Laboratory of Malignant Tumor Epigenetics and Gene Regulation, Sun Yat-Sen Memorial Hospital, Sun Yat-Sen University, Guangzhou, 510120 China; 3 Department of Radiology, The First Affiliated Hospital, Sun Yat-Sen University, No.58 Zhong Shan Road 2, Guangzhou, 510080 China; 4 Department of Radiology, Sun Yat-Sen Memorial Hospital, Sun Yat-Sen University, Guangzhou, 510120 China

**Keywords:** Radiomics, Liver metastasis, Pancreatic ductal adenocarcinoma, Cancer associated fibroblasts

## Abstract

**Background:**

Nearly one fourth of patients with pancreatic ductal adenocarcinoma (PDAC) occur to liver metastasis after surgery, and liver metastasis is a risk factor for prognosis for those patients with surgery therapy. However, there is no effective way to predict liver metastasis post-operation.

**Method:**

Clinical data and preoperative magnetic resonance imaging (MRI) of PDAC patients diagnosed between July 2010 and July 2020 were retrospectively collected from three hospital centers in China. The significant MRI radiomics features or clinicopathological characteristics were used to establish a model to predict liver metastasis in the development and validation cohort.

**Results:**

A total of 204 PDAC patients from three hospital centers were divided randomly (7:3) into development and validation cohort. Due to poor predictive value of clinical features, MRI radiomics model had similar receiver operating characteristics curve (ROC) value to clinical-radiomics combing model in development cohort (0.878 vs. 0.880, *p* = 0.897) but better ROC in validation dataset (0.815 vs. 0.732, *p* = 0.022). Radiomics model got a sensitivity of 0.872/0.750 and a specificity of 0.760/0.822 to predict liver metastasis in development and validation cohort, respectively. Among 54 patients randomly selected with post-operation specimens, fibrosis markers (α-smooth muscle actin) staining was shown to promote radiomics model with ROC value from 0.772 to 0.923 (*p* = 0.049) to predict liver metastasis.

**Conclusion:**

This study developed and validated an MRI-based radiomics model and showed a good performance in predicting liver metastasis in resectable PDAC patients.

**Supplementary Information:**

The online version contains supplementary material available at 10.1186/s13014-023-02273-w.

## Introduction

Pancreatic ductal adenocarcinoma (PDAC) accounts for more than 80% of pancreatic neoplasms and is one of the most lethal malignancies all over the world with a 5-year survival rate of around 8% [1]. Nearly 80% of patients are advanced PDAC when diagnosed and lost the opportunity for operation, and the remaining 20% of patients are feasible for operation as the only potentially curative treatment [2]. However, within patients receiving surgery, parts of patients might experience local recurrence or distant metastasis, even at an early stage. Apart from colorectal cancer, pancreas is the second common original site for liver metastasis [3]. Of note, up to 80% of patients with metastatic pancreatic cancer eventually progress to liver metastasis [4]. Liver metastasis is a strong predictor of poor outcomes of PDAC and the median survival of patients with liver metastasis was reported to be less than 6 months regardless of whether they received resection, palliative therapy or not [5–8]. Meanwhile, for patients receiving operation, 35–50% of patients were observed to get early recurrence within 1 year after surgery, in which nearly 25% of patients occurred to liver metastasis only [9, 10]. Hence, evaluating the risk of liver metastasis after surgery and identifying PDAC patients with high risk are necessary and urgent.

Radiomics were first proposed by Lambin in 2012, which were based on large amounts of high-throughput features extracted from magnetic resonance imaging (MRI) or computerized tomography (CT) images [11]. PDAC is a kind of heterogeneous disease with different clinical behaviors, while radiomics can oversimply the complexities of tumor improvement and behavior [12]. Recently, several researches have revealed the association between radiomics and tumor biology behavior in various malignant diseases, such as tumor phenotype, response to treatment, prognosis and so on [13–16]. Previous study showed that radiomics had a better prediction value for synchronous metastasis and early recurrence in colorectal cancer [17, 18], lung cancer [19] and pancreatic cancer [20–22], which indicated radiomics might be able to help clinical judgment for continuous treatment after surgery. However, while these researches showed clinical application in some occasions, there are no radiomics models reported in current studies to predict liver metastasis post-operation in patients with PDAC.

Meanwhile, cancer associated fibroblasts (CAFs) are the main composition of stroma in pancreatic cancer and take part in fibrosis process in pancreatic cancer [23]. CAFs actively communicate with and stimulate tumor cells, contributing to PDAC development and progression, and recent studies reported CAFs in clinical use associated with PDAC overall survival and lymph node metastasis [24–26]. However, there was no research showed definite association between CAFs and liver metastasis in clinical practice. Radiomics seemed to have a reflection on desmoplasia [27], thus we aimed to evaluate whether fibrosis markers can enhance radiomics model to predict the potential of liver metastasis in PDAC.

In this study, we established and validated an MRI radiomics model to predict the risk of liver metastasis for PDAC patients. Fibrosis markers can promote our risk model but need further validation.

## Method

### Patient population and data management

This study was approved by the Ethics Committee of Sun Yat-sen Memorial Hospital (SYSMH), and patient informed consent was waived for this retrospective research. Between July 2010 and July 2020, a total of 330 patients from SYSMH North District, 201 cases from SYSMH South District (North District and South District) and 251 patients from the First Affiliated Hospital of Sun Yat-Sen University (FAHSYSU) diagnosed as PDAC identified by histopathological examination after upfront surgery were enrolled in this study. The Exclusion criteria included the following: (1) lack of enhanced MRI test within 2 weeks before surgery; (2) low-quality MRI image; (3) history of previous or coexisting other malignant tumors; (4) synchronous liver metastasis (occurrence at base line or within 3 months after surgery); (5) any local or systemic treatment at or before the baseline MRI examination; (6) follow-up for patients without liver metastasis after operation less than 1 year; (7) Patients who died within 3 months after surgery.

Several clinical characteristics, including age, sex, serum tumor biomarkers, and liver function tests were collected before surgery. All pancreas pathological specimens were got from the operation tissue. The pathological stage was referred to the 8th edition American Joint Committee on Cancer (AJCC) [28]. The endpoint of our study was occurrence of liver metastasis after surgery. The positive definition of endpoint were those patients with liver metastasis happened at least 3 months after surgery. Patients identified as non-liver metastasis need at least 1-year follow up to supervise liver metastasis via enhanced CT/MRI scanning.

**Fig. 1:**
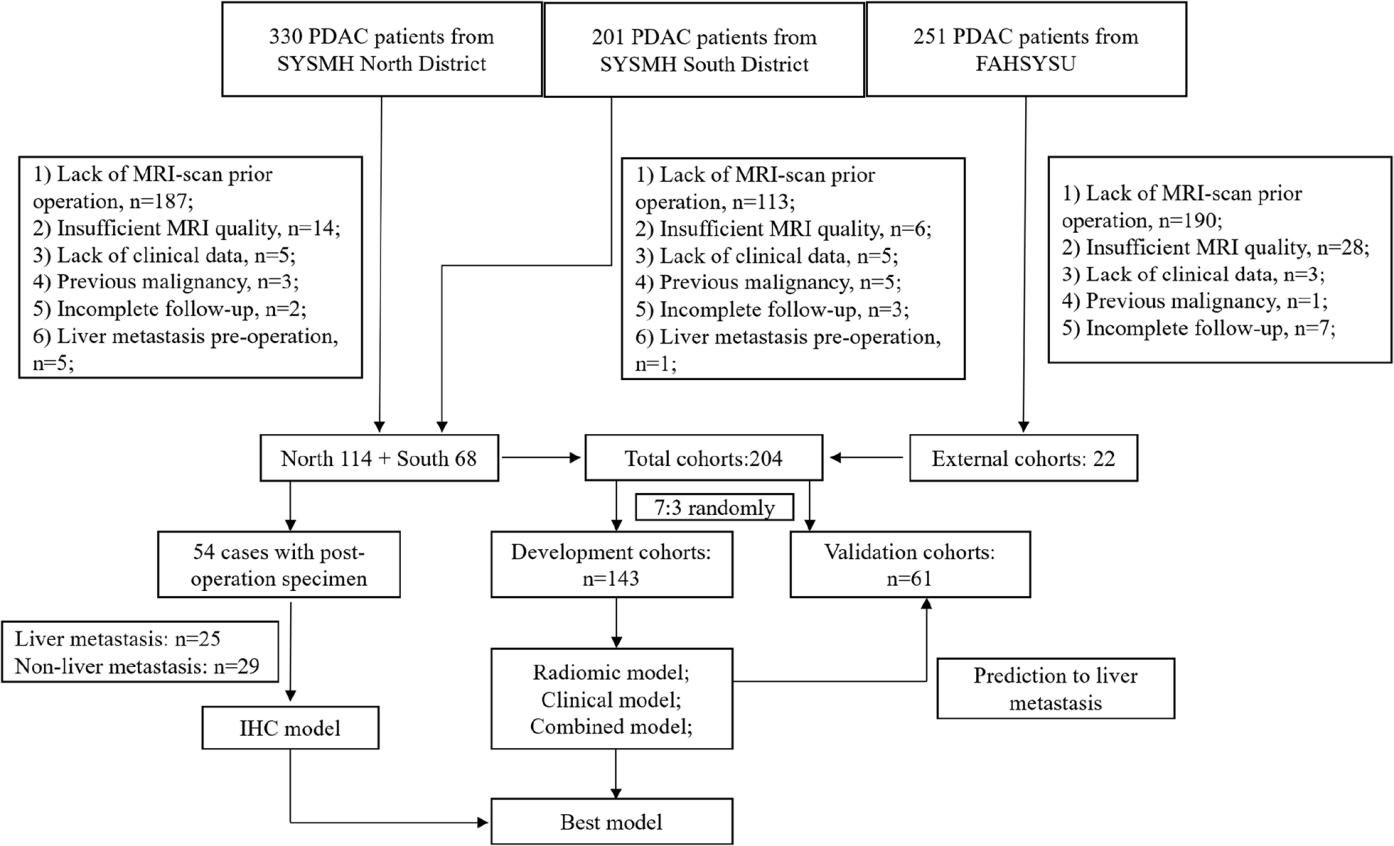
Workflow of this study

Finally, 204 patients were enrolled into the final analysis. Among them, 68 cases came from SYSMH South District, 114 cases came from SYSMH North District, and 22 cases were from FAHSYSU. Patients from three centers were divided randomly (7:3) into the development (n = 143, 70.1%) and validation (n = 61, 29.9%) cohort (Fig. 1). Meanwhile, we randomly chose 54 patients, including 29 cases with liver metastasis and 25 cases without liver metastasis, from SYSMH who had pancreas pathological specimens after operation to test CAFs markers in primary tumor by immunohistochemistry (IHC) test.

### Imaging acquisition

All imaging data included three sets of images from three different scanners: preoperative T1w (weight), T2w, and contrast enhancing T1w (CE-T1w) portal venous phase MR images. T2w was respiratory triggering and CE-T1w was performed following the intravenous injection of 0.1 mmol/kg contrast medium (Gadodiamide) as rate = 2 mL/s. The portal venous phase (60 s post-injection) was collected. The details of MRI protocol were shown in Additional file 1: Table S1.

**Fig. 2:**
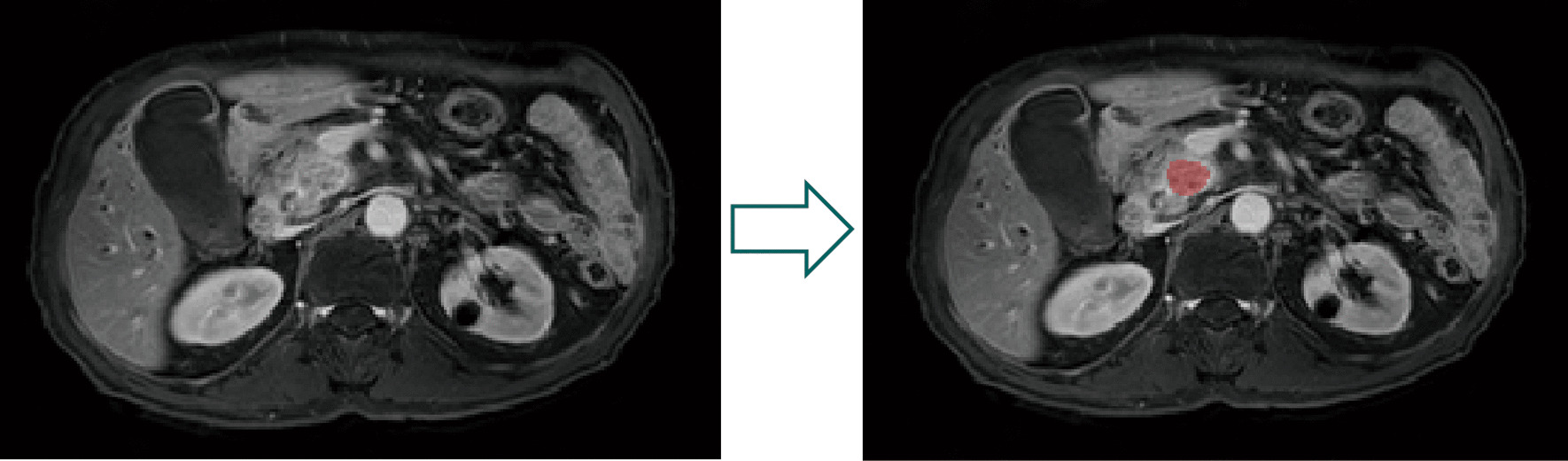
Manual segmentation of region of interest in pancreatic cancer

Preoperative T1w, T2w, and CE-T1w imaging were used to extract radiomics features. The MRIcroGL software (version 1.2.20210317, https://www.nitrc.org) was used to transfer the original DICOM images to the NIFTI format before the segmentation of regions of interests (ROIs). The ROIs were manually contoured by two radiologists (G. M and L. YJ), who both had more than 10-year experience of interpreting abdominal images and were blinded to the clinical outcome during the ROI segmentation. All ROI work was finished by ITK-SNAP v.3.8.0 from UPenn (www.itksnap.org) [29] (Fig. 2). All images were normalized as 100 bin width scale and then resampled to voxels of 3 × 3 × 3 mm by sitkBSpline method of interpolator. Subsequently, 1302 high-throughput radiomics features for each MRI sequence were automatically extracted from the platform based on the “Pyradiomics” package in Python (version 3.10.1, https://pyradiomics.readthedocs.io) [30]. The radiomics features were classified into following four groups: (a) First-order statistics (n = 18); (b) shape features (n = 14); (c) Texture features, including gray level co-occurrence matrix (GLCM, 23 features), gray level run length matrix (GLRLM, 16 features), gray level size zone matrix (GLSZM, 16 features), neighboring gray tone difference matrix (NGTDM, 5 features) and gray level dependence matrix (GLDM, 14 features); (d) higher-order statistical features (n = 1196) consisting of fist-order statistics, shape features and texture features derived from wavelet filter and Laplacian of Gaussian filter (σ-1, 2, 3, 4, 5).

### Development of radiomics signatures

Before screening radiomics features, all features were normalized by Z-score normalization. Least absolute shrinkage and selector operation (LASSO), random forest (RF) algorithm (the top 20 candidates), and principal component analysis (PCA) were used to filter the most useful radiomics features from high-dimensional imaging data from T1w, T2w, and CE-T1w images. Then supporting vector machine (SVM) model and logistic regression (LR) model were used to establish prediction models. The best model to predict liver metastasis was selected among LASSO-SVM, LASSO-LR, RF-SVM, RF-LR, PCA-SVM, and PCA-LR models.

### Immunohistochemistry

The tissue after surgery was embedded in paraffin and sectioned into 4 μm-thick slices for immunohistochemical staining. The sections were incubated with primary antibody (α-smooth muscle actin, α-SMA, *proteintech 14,395–1-AP*) according to antibody specification. The IHC staining scores were assessed depending on the staining intensity which were graded on scale from 0 to 3 (0, 0–25%; 1, 26–50%; 2, 51–75%; 3, 76–100%).

### Comparison of risk models

Univariate logistic regression was applied to the development cohort for each clinical characteristic to choose the independent clinical features. The significant variables (*p* < 0.05) were then included in multivariable logistic regression analysis to build clinical prediction model for liver metastasis. Afterward, significant radiomics signatures and clinical characteristics were included in multivariable logistic regression to establish a combined model to predict liver metastasis in the development cohort. Then combined model, radiomics model and clinicopathological model were compared via area under the curve (AUC) of receiver operating characteristic (ROC) curve analysis in the development and validation cohort. The decision curve analysis (DCA) was used to calculate the net benefits to estimate the clinical utility of forecasting model. Meanwhile, we used IHC specimen cohort to evaluate whether the CAFs markers could make our predictive model much more suitable via comparing ROC curve and DCA.

### Statistical analysis

Continuous variables are expressed as mean ± standard deviation (SD), while categorical variables are shown as frequency and percentage. The independent *t*-test and Mann–Whitney *U*-test were used to compare continuous variables, while the chi-squared test or Fisher’s exact test were used to analyze the categorical variables when necessary. Delong’s test was applied to compare AUC of ROC curve.

All the work for significant variables screening, model establishment, and model comparison were finished by R software (version 4.1.2, https://www.r-project.org/). SPSS 22.0 was applied to perform the statistical analysis. All statistical tests were two-sided with statistical significance accepted at *p* < 0.05.

## Results

### Clinical characteristics

In total, 204 PDAC cases from three hospital centers in China were included in this study. Among them, 68 cases came from SYSMH South District, 114 cases came from SYSMH North District, and the other 22 cases were from FAHSYSU (Fig. 1). The baseline characteristics of development cohort and validation cohort were shown in Table 1. No significant differences between development and validation cohort were detected. During median 12 (3–89) month follow-up, 55 patients (22.7%) got liver metastasis identified by following enhanced MRI or CT detection and the median time of liver metastasis occurrence was 6 (3–17) months after surgery.

**Table 1:** Clinicopathological characteristics of patients in development and validation cohorts

Characteristics	Total cohort	Development cohort	Validation cohort	*p*
Age (y)	60.5 ± 10.0	59.8 ± 10.0	62.1 ± 9.8	0.129
Gender				0.703
Male (%)	81 (39.7%)	58 (40.5%)	23 (37.7%)	
Female (%)	123 (60.3%)	85 (59.5%)	38 (62.3%)	
Tumor biomarker pre-operation				
CA199 (U/ml)	964.4 ± 2683.3	1087.5 ± 2961.0	675.9 ± 1868.1	0.317
CEA (mg/ml)	7.6 ± 16.8	7.4 ± 15.2	8.1 ± 20.2	0.792
AFP (IU/ml)	3.6 ± 3.8	3.7 ± 4.4	3.2 ± 1.9	0.399
Tumor region				0.711
Head	164 (80.4%)	114 (79.7%)	50 (82.0%)	
Body/tail	40 (19.6%)	29 (20.3%)	11 (18.0%)	
Operation method				0.865
Partial resection	13 (6.4%)	10 (7.0%)	3 (4.9%)	
PD	153 (75.0%)	107 (74.8%)	46 (75.4%)	
PPPD	2 (1.0%)	1 (0.7%)	1 (1.6%)	
Distal pancreatic resection	36 (17.6%)	25 (17.5%)	11 (18.1%)	
T stage				0.370
T1-2	90 (44.1%)	66 (46.2%)	24 (39.3%)	
T3	114 (55.9%)	77 (53.8%)	37 (60.7%)	
N stage				0.242
0	93 (45.6%)	69 (48.3%)	24 (39.3%)	
1–2	111 (54.4%)	74 (51.7%)	37 (60.7%)	
Differentiation (poorly, %)	63 (30.9%)	41 (28.7%)	22 (36.1%)	0.295
PNI (%)	147 (72.1%)	102 (71.3%)	45 (73.8%)	0.722
Surgical margin (R1, %)	17 (8.3%)	14 (9.8%)	3 (4.9%)	0.249
Post-operation chemotherapy	103 (50.5%)	75 (52.4%)	28 (45.9%)	0.392
Liver metastasis (%)	55 (22.7%)	39 (27.3%)	16 (26.2%)	0.878
Time for liver metastasis after surgery (month)	6 (3–17)	3 (3–17)	6 (3–13)	0.498
Follow-up time (month)	12 (3–89)	12 (3–89)	12 (3–34)	0.179

*PD* Pancreaticoduodenectomy, *PPPD* Pylorus-preserving pancreaticoduodenectomy, *PNI* Perineural invasion

### Radiomics signature for liver metastasis prediction

**Fig. 3:**
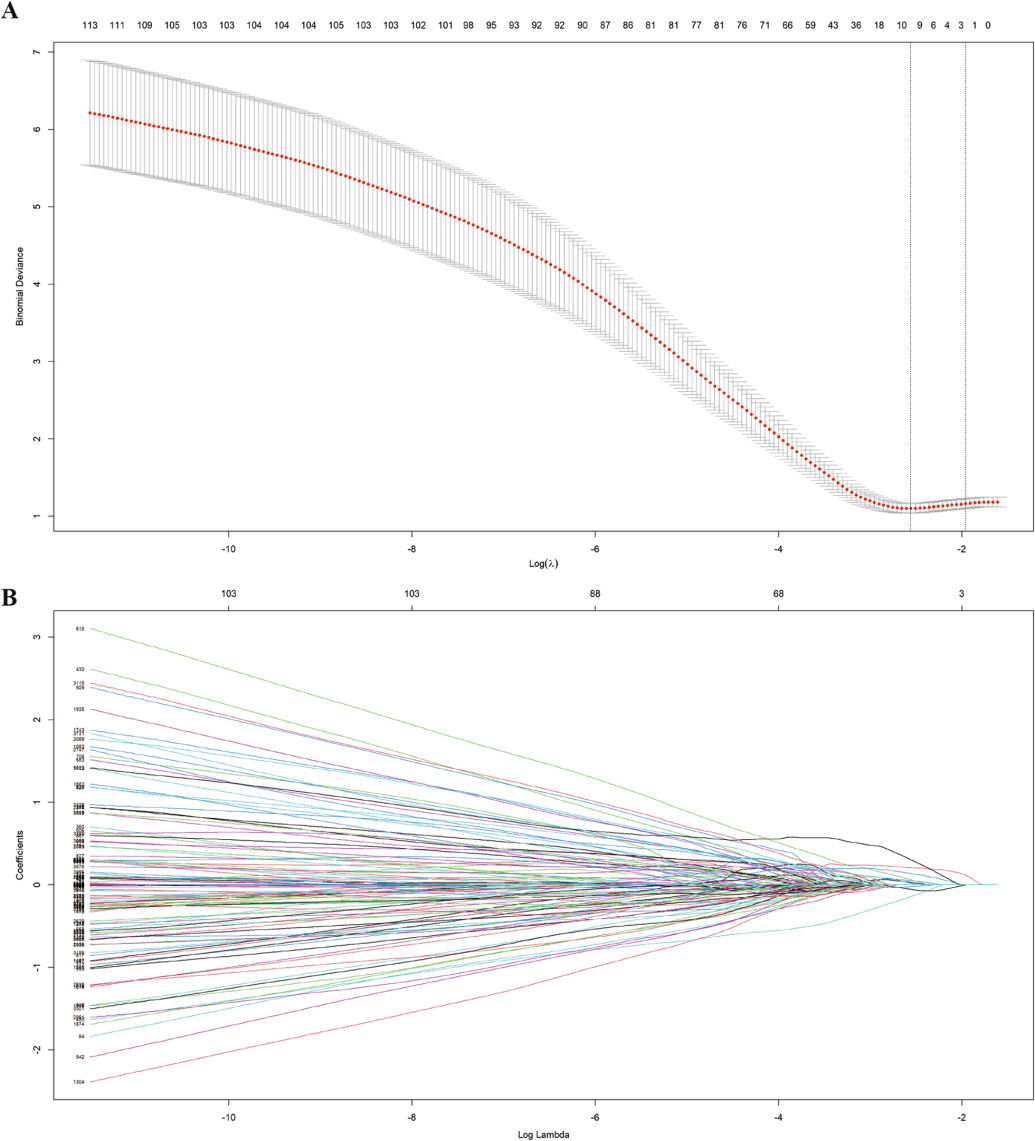
LASSO regression for radiomics features selection. **A** In the LASSO model, tenfold cross-validation as the minimum criteria was used to select the penalization parameter λ. The minimum criteria and the 1-SE criteria were used to draw the dotted vertical lines at the optimal values. **B** The illustration about LASSO coefficient profiles to prediction for liver metastasis in pancreatic cancer patients

3906 radiomics features from T1w, T2w, and CE-T1w were used to establish a prediction model. In the development and validation cohort, the LASSO-SVM model resulted in AUCs of 0.878 and 0.815, respectively; the LASSO-LR model got AUCs of 0.870 and 0.821, respectively; the RF-SVM model calculated the AUCs as 0.788 and 0.736, respectively; the RF-LR model resulted in AUCs of 0.840 and 0.710, respectively; the PCA-SVM model leaded to AUCs of 0.794 and 0.756, respectively; and the PCA-LR model induced the AUCs of 0.825 and 0.762, respectively (Additional file 1: Table S2). Though LASSO-SVM model had similar AUC to LASSO-LR model, it had better sensitivity/specificity in development and validation cohort (0.872/0.760 vs. 0.949/0.654, and 0.750/0.822 vs. 0.250/0.956, respectively). Therefore, the LASSO-SVM model was selected as the most suitable model with the best performance to predict liver metastasis. The results of LASSO regression were shown in Fig. 3 and 10 variables included in LASSO-SVM model were displayed in Additional file 1: Table S3. Based on the output probability of the SVM model, we convert the output probability into radiomic score (Rad score = P_SVM_, range 0–1, OR 3780, 95% CI 286–84,293, *p* < 0.001).

### Clinical model for liver metastasis prediction

The association between clinical characteristics and liver metastasis in development cohort was shown in Table 2. The variables with p value less than 0.05 in univariable analysis were considered for clinical prediction model. As a result, age (OR 0.946, *p* = 0.007) and N stage (OR 3.209, *p* = 0.004) were included for Logistic regression model. Finally, the clinical model consisted of age and N stage resulted in an AUC of 0.712 and 0.551 in development and validation cohort, respectively.

**Table 2:** Univariate and multivariate analysis of clinical characteristics associated with liver metastasis in development cohort

Characteristics	Univariate logistic regression		Multivariate logistic regression	
	OR (95% CI)	*p*	OR (95% CI)	*p*
Age (y)	0.946 (0.907–0.983)	0.007	0.951 (0.911–0.990)	0.018
Gender (male/female)	1.31 (0.62–2.86)	0.487		
Tumor biomarker pre-operation				
CA199 (U/ml)	1.0001(0.9999–1.0002)	0.229		
CEA (mg/ml)	1.001 (0.969–1.025)	0.919		
AFP (IU/ml)	1.087 (0.999–1.230)	0.091		
Tumor region (head/body + tail)	0.648 (0.274–1.598)	0.331		
T stage (T3/T1–2)	0.867 (0.410–1.815)	0.706		
N stage (N1-2/N0)	3.209 (1.478–7.371)	0.004	2.883 (1.303–6.718)	0.011
Differentiation (well/poorly)	0.626 (0.286–1.396)	0.244		
PNI ( ±)	1.481 (0.649–3.635)	0.367		
Surgical margin (R1/R0)	1.074 (0.280–3.445)	0.909		
Post-operation chemotherapy ( ±)	1.663 (0.791–3.581)	0.184		

*PNI* Perineural invasion

**Fig. 4:**
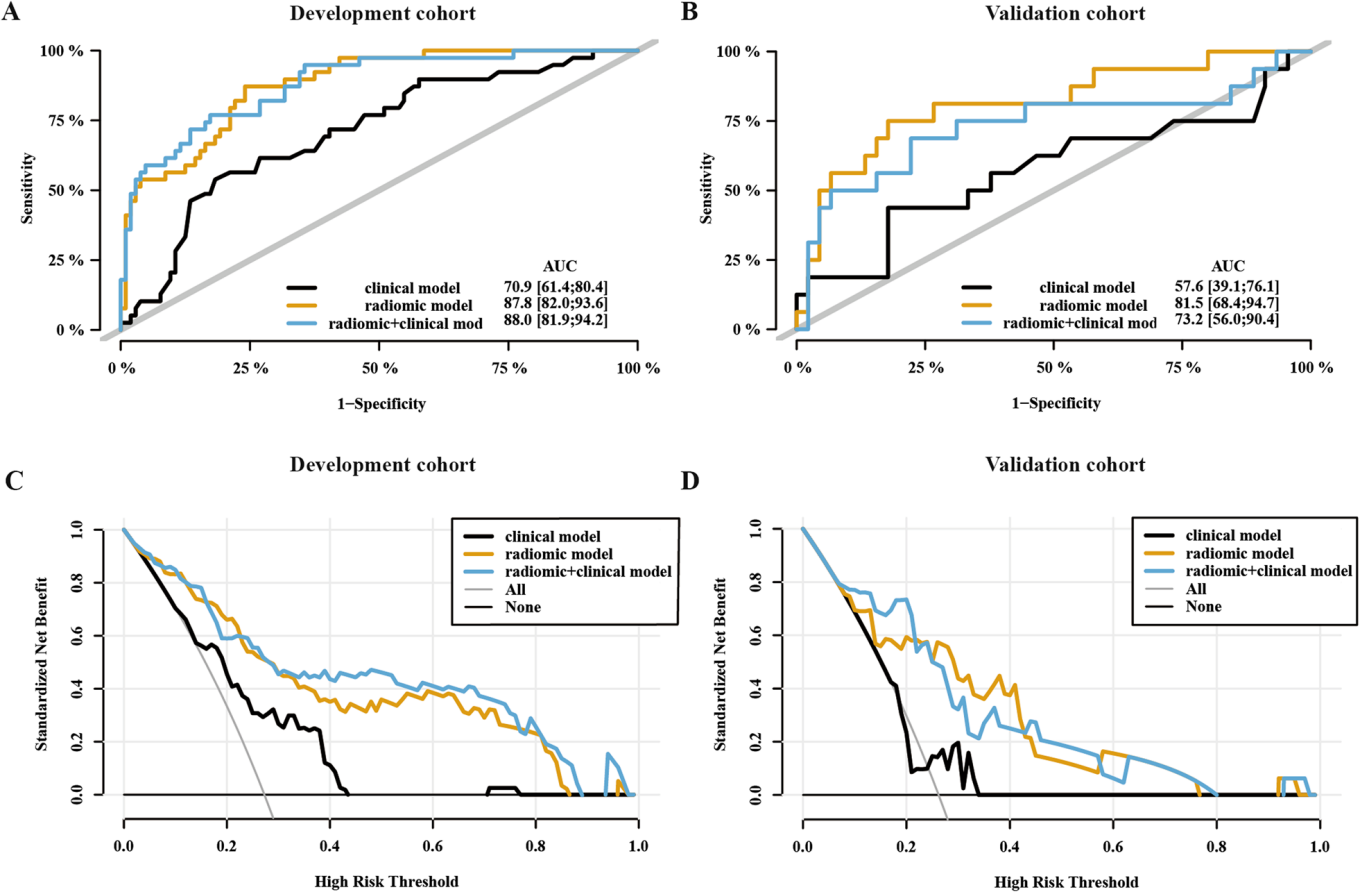
The ROC curve and decision curve of prediction model in development and validation cohort. Discriminatory accuracy in predicting liver metastasis was detected by ROC analysis for comparing the AUC in development (**A**) and validation (**B**) cohorts. Decision curve analysis for combined, radiomic, clinical model in development (**C**) and validation (**D**) cohorts

The multivariable logistic regression was used to combine clinical signatures and radiomics signatures to predict liver metastasis. The clinical-radiomics model and radiomics model had similar AUC value in development cohort (0.880 vs. 0.878, *p* = 0.897), both of which were better than single clinical model in development cohort (0.880 vs. 0.709, *p* < 0.001; 0.878 vs. 0.709, *p* = 0.002, respectively). Due to poor prediction value of clinical model in validation cohort (AUC = 0.576), the AUC of combing model was worse than single radiomics model in validation cohort (0.732 vs. 0.815, *p* = 0.022, Fig. 4). The DCA curve also showed radiomics model provided a better net benefit of threshold probabilities to predict liver metastasis than combination model due to poor net benefit of clinical model in validation cohort (Fig. 4c, d). Due to poor ROC value of combination model Finally, we chose radiomics model alone as the best prediction model to predict the risk of liver metastasis in pancreatic cancer after surgery.

### *IHC model *via* CAFs biomarker promoting prediction value of radiomics model*

**Fig. 5:**
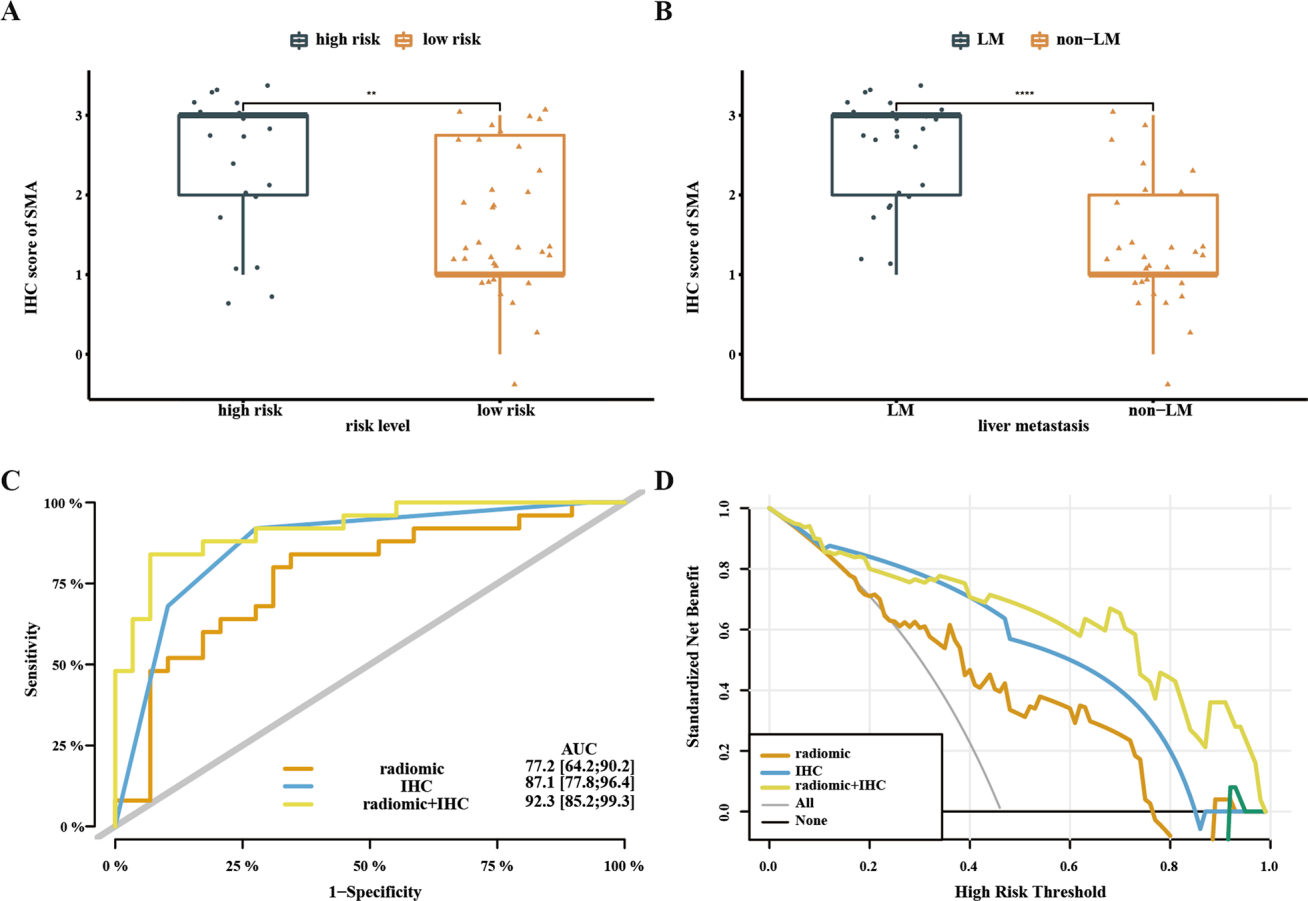
IHC score of α-SMA in PDAC promoting radiomics model. **A** α-SMA level in different risk group by radiomics score. **B** α-SMA score in PDAC patients with/without liver metastasis. **C** ROC curve of different risk models in PDAC patients with IHC staining. **D** The decision curve of different risk models in PDAC patients with IHC staining

Because clinical model provided a deplorable prediction value for liver metastasis after surgery, we had to consider the value of pathological staining for these patients receiving operation. To evaluate the predictive value of fibrosis in liver metastasis, we randomly chose 54 patients from the internal cohort with post-operation pancreas specimens (25 liver metastasis vs. 29 non-Liver metastasis) and took IHC test for CAFs markers. We selected α-SMA as a representative marker for fibrosis and the detailed standards of IHC scores were shown in Additional file 1: Fig. S1. For patients with a high risk of liver metastasis via radiomics score (Rad score > 0.362), they got a much higher IHC score of α-SMA than those with low risk (2.35 vs. 1.65, *p* = 0.010, Fig. 5a). Similar results were shown in patients with liver metastasis compared with those without liver metastasis (2.36 vs. 1.31, *p* < 0.001, Fig. 5b). Then we evaluated our risk model in this cohort. The radiomics and IHC combination model provided better AUC (0.923 vs. 0.772, *p* = 0.049) and decision curve than single radiomics model, but no difference of AUC value was found between radiomics model and IHC model (0.772 vs. 0.871, *p* = 0.246, Fig. 5c, d).

## Discussion

Liver metastasis is a common and serious outcome for PDAC patients, and patients relapsed with liver metastasis had a worse prognosis compared those without recurrence [8]. Thus, we hoped to establish a prediction system to forecast the risk of liver metastasis for those PDAC patients receiving surgery in early stage, in order to carry out more aggressive intervention therapy and follow-up. In this multicenter study, we established and validated an MRI-based radiomics model to predict liver metastasis in individuals with PDAC after operation. We extracted high-throughput radiomics features from T1w, T2w and CE-T1w sequence of MRI images before operation, and developed a prediction model for liver metastasis in development and validation cohort from three hospital centers.

In radiomics features, histogram feature is a low-order radiomics parameter, describing the distribution of voxel intensities related to the properties of individual pixels [31]. As previous study reported, some histogram features were correlated to the biobehavioral, like hypoattenuation value reflecting degree of tumor necrosis and isoattenuating enhancement pattern meaning probably well-differentiated tumor [32, 33]. However, shape features and histogram features showed no prediction value for liver metastasis and did not included in our finally model. For texture features, especially some high-order features derived from various transform filter, showed well prognosis value in various malignancies, including breast cancer, colorectal cancer, pancreatic cancer and so on [34–36]. Our results proved that high-order features had a well prediction value for liver metastasis in PDAC patients, indicating their value reflecting to tumor behavior process, which was also verified in researches utilizing texture signatures to predicting early recurrence in PDAC [20]. However, high-order texture features had their own problems that they could not be easily interpreted in clinical description. Due to challenges to associate single radiomics signature with complex tumor biological behaviors, a multifactor model formed from various radiomics features can ameliorate this issue. Our results showed that the radiomics model derived from development cohort achieved a satisfactory prediction value for liver metastasis in validation cohort as well.

In our analysis, age was a protection factor for liver metastasis and older patients were much easier to occur to liver metastasis. Patients with lymph node metastasis seemed much easier to occur to liver metastasis after surgery, which were not confirmed in our validation cohort. Though PDAC with high tumor size (T stage) or lymph node metastasis had higher likelihood of metastasis, patients including in our analysis had no advanced metastasis before surgery and most of them received complete R0 resection, which is the possible reason why tumor size and lymph node metastasis had worse prediction value in our cohort. Previous study also reported tumor region and extra-pancreatic neuropathy might had association with liver metastasis post-operation [37, 38]. They showed tumors in pancreatic body or trail were easier to metastasis but not verified in our study. 72.1% cases in our cohort had PNI and our data showed no correlation between PNI and liver metastasis. However, no research proved that any useful prediction model based on such clinicopathological features were established or validated. Meanwhile, in our study, the clinicopathological features also had poor prediction value to liver metastasis, which was not consistent in validation cohort. Further study should pay more attention to exploring much more effective clinical features to predict risk of liver metastasis in pancreatic cancer. A good example was that, Tien et al. [39] proposed that a high circulating tumor cells (CTC) in portal vein had a 64.7% sensitivity and 95.4% specificity to predict liver metastasis within 6 months after surgery, which needed further validation.

Due to the poor predictive value of clinical characteristics for liver metastasis in PDAC patients receiving surgery, we tried to use pathological staining to enhance our radiomics model. Compared with clinical characteristics, pathological signatures seemed to have a better ability to reflect bio-behaviors of PDAC. Pancreatic cancer is rich in fibrous stroma and CAFs are the main cells mostly from mesenchymal origin found in tumor microenvironment playing an important role in fibrosis [40]. Besides metastasis foci, CAFs can also promote primary tumor cells invasion and metastasis [41]. Sugai et al. [42] reported CAFs might have a prediction value of lymph node metastasis in invasive submucosal colorectal cancer, and CAFs with nectin-1, monocarboxylate transporters (MCTs), fibroblast activation protein (FAP) marker had prediction value of lymph node metastasis or overall survival in pancreatic cancer [24, 43, 44], suggesting potential clinical use of CAFs in prognosis. In our study, we found radiomics signature had a good association with fibrosis level in pancreatic cancer, and the addition of α-SMA score promoted AUC of radiomics model. Wang et al. [25] pointed pancreatic cancer with loose-type had a higher potential of metastasis, but our results suggesting dense-type were easier to occur to liver metastasis. However, α-SMA is a common marker of CAFs while the consists of CAFs had heterogeneity [40, 45]. Different subtype of CAFs had their own function and markers, and the heterogeneity of CAFs influenced the potential of liver metastasis in individual patient. Therefore, further study should concentrate on the composition of subtype of CAFs to evaluate the risk of liver metastasis. Our study provided a potential for fibrosis level to predict liver metastasis for PDAC patients receiving surgery, and further study can consider MR scanning for pancreas stiffness detection to replace of IHC staining in order to neoadjuvant treatment to reduce risk of liver metastasis after surgery.

However, there are still several limitations in our study. First, it was a retrospective study with heterogeneity in MR acquisition among different MR scanners between three different hospital centers. Therefore, we made normalization and resample before radiomics features extraction to reduce heterogeneity, and further work might concentrate on a prospective verification of our risk model based on standard MR scanning protocol and unified MR scanner. Second, the cases with specimen for IHC analysis were limited and much CAFs associated marker were also considered to including in further risk model. Third, due to the limitation of amounts of patients in three hospital centers, we mixed patients and divided into development and validation cohorts, and further study should include more patients from different centers to validate our model.

In conclusion, this research established and validated a radiomics model to predict liver metastasis after surgery in PDAC patients. This prediction model can assist clinicians to decide clinical treatment after operation for patients with high risk of liver metastasis. CAFs markers has their own potential to predict liver metastasis combining with radiomics, but still need a further validation.

## Supplementary Information

**Additional file 1: Table S1**. MRI scanning parameters for patients. **Table S2**. AUC of ROC analysis in different risk model for liver metastasis prediction. **Table S3.** Variables of radiomic features selected by LASSO regression. **Fig. S1.** Standard figures of α-SMA IHC scores.

## Data Availability

The datasets used and/or analyzed during the current study are available from the corresponding author upon reasonable request.

## Abbreviations

PDAC: Pancreatic ductal adenocarcinoma
MRI: Magnetic resonance imaging
ROC: Receiver operating characteristics
CAFs: Cancer associated fibroblasts
IHC: Immunohistochemistry
CE-T1w: Contrast enhancing T1w
ROIs: Regions of interests
GLCM: Gray level co-occurrence matrix
GLRLM: Gray level run length matrix
GLSZM: Gray level size zone matrix
NGTDM: Neighboring gray tone difference matrix
GLDM: Gray level dependence matrix
LASSO: Least absolute shrinkage and selector operation
RF: Random forest
PCA: Principal component analysis
SVM: Supporting vector machine
LR: Logistic regression
AUC: Area under the curve
DCA: Decision curve analysis
SD: Standard deviation
PNI: Perineural invasion
α-SMA: α-Smooth muscle actin
