# Supplementary material for: Development and validation of a radiomics model of magnetic resonance for predicting liver metastasis in resectable pancreatic ductal adenocarcinoma patients
Source: Radiat Oncol. 2023 May 10;18:79. doi: 10.1186/s13014-023-02273-w (PMC10170860; doi:10.1186/s13014-023-02273-w)
Supplement: Supplementary file 1 — Additional file 1: Table S1. MRI scanning parameters for patients. Table S2. AUC of ROC analysis in different risk model for liver metastasis prediction. Table S3. Variables of radiomic features selected by LASSO regression. Fig. S1. Standard figures of α-SMA IHC scores. [file 13014_2023_2273_MOESM1_ESM.docx]

Supplementary Table 1. MRI scanning parameters for patients

| Hospital | Scanner | Sequence | TR/TE (ms) | FOV  (mm) | Matrix | Slice thickness (mm) | Slice Gap  (mm) | Slices | Flip  Angle | Acquisition Time (min) |
| --- | --- | --- | --- | --- | --- | --- | --- | --- | --- | --- |
| SYSMH | Skyra | T1-w | 3.97/1.26 | 400 | 204*320 | 2 | 0.4 | 88 | 9 | 16s |
|  |  | T2-w | 3000/89 | 380 | 204*320 | 4 | 1 | 35 | 108 | 206s |
|  |  | T1+C | 3.97/1.26 | 400 | 204*320 | 2 | 0.4 | 88 | 9 | 16s |
|  | Vida | T1-w | 4.03/1.28 | 400 | 320*320 | 2.5 | 0.5 | 88 | 11 | 15s |
|  |  | T2-w | 3000/82 | 380 | 320*320 | 5 | 1 | 40 | 110 | 95s |
|  |  | T1+C | 4.03/1.28 | 400 | 320*320 | 2.5 | 0.5 | 88 | 11 | 15s |
| FAHSYSU | Siemens-prisma | T1-w | 3.84/1.45 | 380 | 187*288 | 2 | 0.4 | 96 | 25 | 16s |
|  |  | T2-w | 2000/77 | 380 | 288*384 | 5 | 1 | 30 | 103 | 180 |
|  |  | T1+C | 2.75/1.05 | 380 | 192*320 | 2 | 0.4 | 96 | 12.5 | 35s |

Supplementary Table 2. AUC of ROC analysis in different risk model for liver metastasis prediction

| **AUC** | **PCA-SVM** | **PCA-LR** | **LASSO-SVM** | **LASSO-LR** | **RF-SVM** | **RF-LR** |
| --- | --- | --- | --- | --- | --- | --- |
| Developing cohort | 0.794 | 0.825 | 0.878 | 0.870 | 0.788 | 0.840 |
| Validation cohort | 0.756 | 0.762 | 0.815 | 0.821 | 0.736 | 0.710 |

Supplementary Table 3. Variables of radiomic features selected by LASSO regression.

| Radiomic features |
| --- |
| T1W_log.sigma.1.0.mm.3D_glszm_LowGrayLevelZoneEmphasis |
| T1W_log.sigma.4.0.mm.3D_glszm_SmallAreaEmphasis |
| T1W_log.sigma.5.0.mm.3D_glrlm_ShortRunLowGrayLevelEmphasis |
| T1W_wavelet.LHL_glrlm_LowGrayLevelRunEmphasis |
| T1W_wavelet.LHL_glszm_ZoneEntropy |
| T1W_wavelet.LHH_glszm_LowGrayLevelZoneEmphasis |
| T1W_wavelet.HLL_glszm_LowGrayLevelZoneEmphasis |
| T1W_wavelet.HHL_glrlm_ShortRunLowGrayLevelEmphasis |
| T1W_wavelet.LLL_glrlm_RunEntropy |
| T2W_log.sigma.4.0.mm.3D_glcm_InverseVariance |


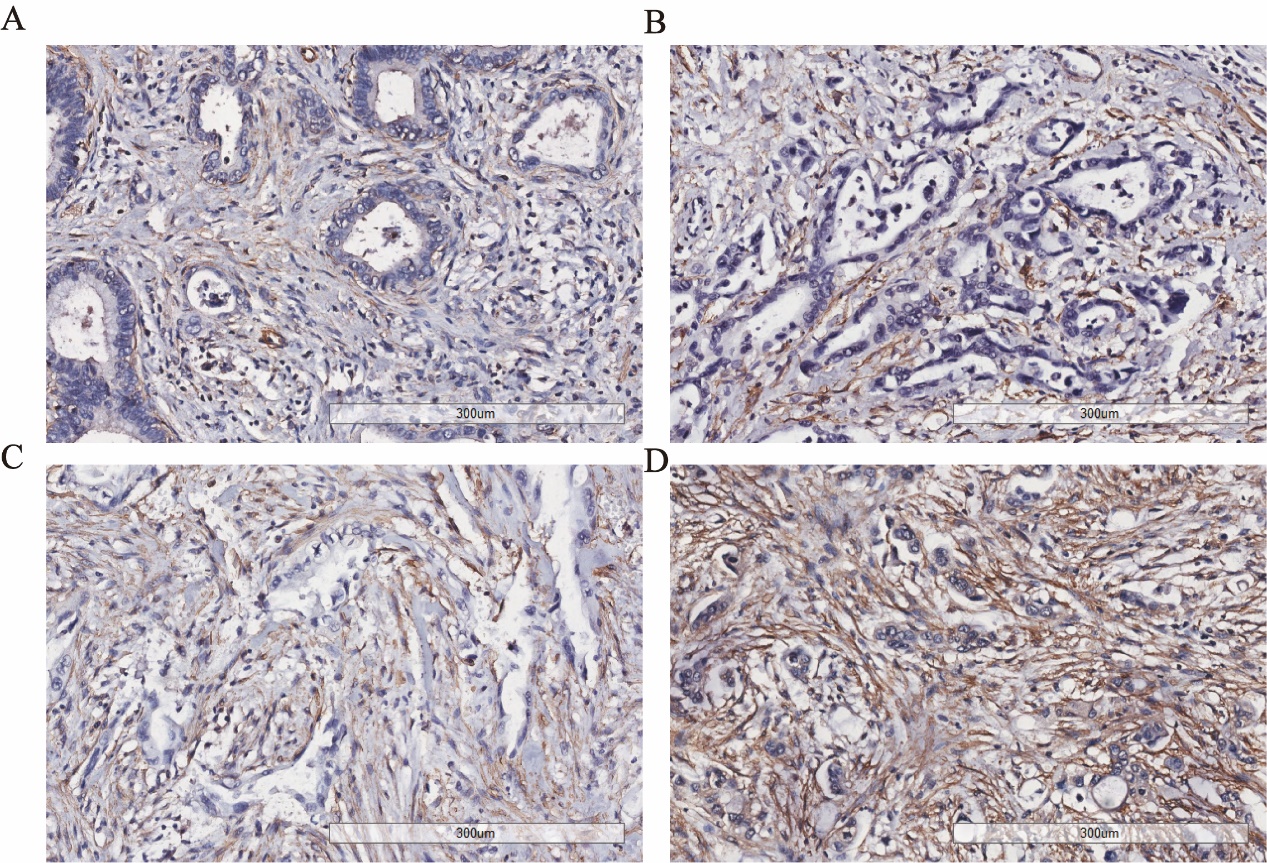


Supplementary Figure 1. Standard figures of α-SMA IHC scores. Representative illustrations of PDAC with α-SMA staining of 0, 1, 2 and 3 score (A-D).
